# Placental and Breast Metastasis of Squamous Cell Carcinoma in a Patient With Recurrent Respiratory Papillomatosis

**DOI:** 10.7759/cureus.46183

**Published:** 2023-09-29

**Authors:** Venumadhavi Gogineni, Luke Gingell, Merryl Varghese, Borys Hrinczenko

**Affiliations:** 1 Internal Medicine, Michigan State University, East Lansing, USA; 2 Internal Medicine, Michigan State University- Sparrow Hospital, Lansing, USA; 3 Oncology, Michigan State University College of Human Medicine, East Lansing, USA; 4 Oncology, Michigan State University, East Lansing, USA

**Keywords:** cancer in pregnant patients, breast metastasis of non-mammary tumors, placental metastasis, squamous cell lung carcinoma, recurrent respiratory papillomatosis

## Abstract

Recurrent respiratory papillomatosis (RRP), which is usually benign, is an intractable disease characterized by recurrent papillomas (wart-like lesions). Although it most commonly involves the mucosal epithelial lining of the upper respiratory tract, on rare occasions, it can also involve lung parenchyma. RRP carries the risk of malignant transformation, most often to non-small-cell squamous lung cancer. Here, we present the case of a 32-year-old pregnant female with a past medical history of RRP who developed mild respiratory distress during her immediate postpartum period. This prompted imaging of the chest which revealed right lower lobe hypodensities with extensive hilar and perihilar lymphadenopathy. Histopathology of the bronchial specimen showed squamous cell carcinoma with 100% programmed death-ligand 1 (PD-L1) expression. Gross examination of the patient’s placenta showed multiple tan-colored nodules which was confirmed on histopathological examination as multifocal regions of squamous cell carcinoma metastatic from the lung. The patient underwent a staging positron emission tomography (PET) scan which showed hypermetabolic regions in the right middle and lower lobes of the lung, with avidity in the right paratracheal region and an enhancing lesion in the left breast. Biopsy from the breast lesion was also positive for squamous cell carcinoma and PD-L1. She was diagnosed with Stage IVB (T1c, N3, M1c) non-small-cell squamous lung cancer and was started on pembrolizumab. Carboplatin and paclitaxel were added after an initial mixed response to therapy. The patient was non-compliant with her updated treatment regimen as well as with outpatient follow-up visits. A restaging PET scan demonstrated an inadequate response to the amended immunotherapy/chemotherapy regimen. Ultimately, she passed away within one and a half years of her initial diagnosis. Malignant transformation of papillomatous lesions into squamous cell cancer is infrequent, and the occurrence of metastasis to the breast and/or placenta is exceptionally rare. To our knowledge, this is the first reported case of placental and breast metastasis of squamous cell lung cancer in a patient with RRP.

## Introduction

Recurrent respiratory papillomatosis (RRP) is a rare disease that presents as benign and recurrent exophytic lesions involving the mucosal lining of the aero-digestive tract. While RRP is usually confined to the upper respiratory tract, distal spread occurs in 5-48% of cases, with 1% of cases involving the lung parenchyma [1-4]. While uncommon, lung involvement is associated with an aggressive disease course, often requiring repeated hospitalizations for the management of airway obstruction and infection [5,6]. RRP primarily afflicts two populations: children and young adults. Most cases of juvenile-onset RRP (JoRRP) occur through vertical transmission of human papillomavirus (HPV) serotypes 6 and 11, harboring a relatively low risk of malignant transformation compared to other HPV serotypes. However, the risk of transformation is increased in patients with prior tobacco use [7,8]. Here, we present the case of a 32-year-old female with a past medical history of JoRRP and tobacco use who developed placental and breast metastases following malignant transformation of RRP to squamous cell carcinoma during pregnancy.

## Case presentation

The patient is a 32-year-old postpartum female with a past medical history of tobacco use since age 13 and JoRRP involving the nose, larynx, and trachea (diagnosed at age two). Her respiratory lesions had been previously managed by laser therapy and excision. The patient initially presented to the emergency department at 39 weeks gestation with intensifying contractions. After an artificial rupture of membranes, the patient underwent an uneventful vaginal delivery the following day. On postpartum day one, the patient developed difficulty breathing with associated wheezing and dry cough. Further interview revealed that the patient’s cough and difficulty breathing had been present throughout pregnancy and were progressively worsening. The patient had been seen in the emergency department for these symptoms and treated for pneumonia multiple times. She also endorsed unintentional weight loss during pregnancy. Her RRP lesions had been appropriately followed with her last lesion excision occurring two months before parturition.

**Figure 1:**
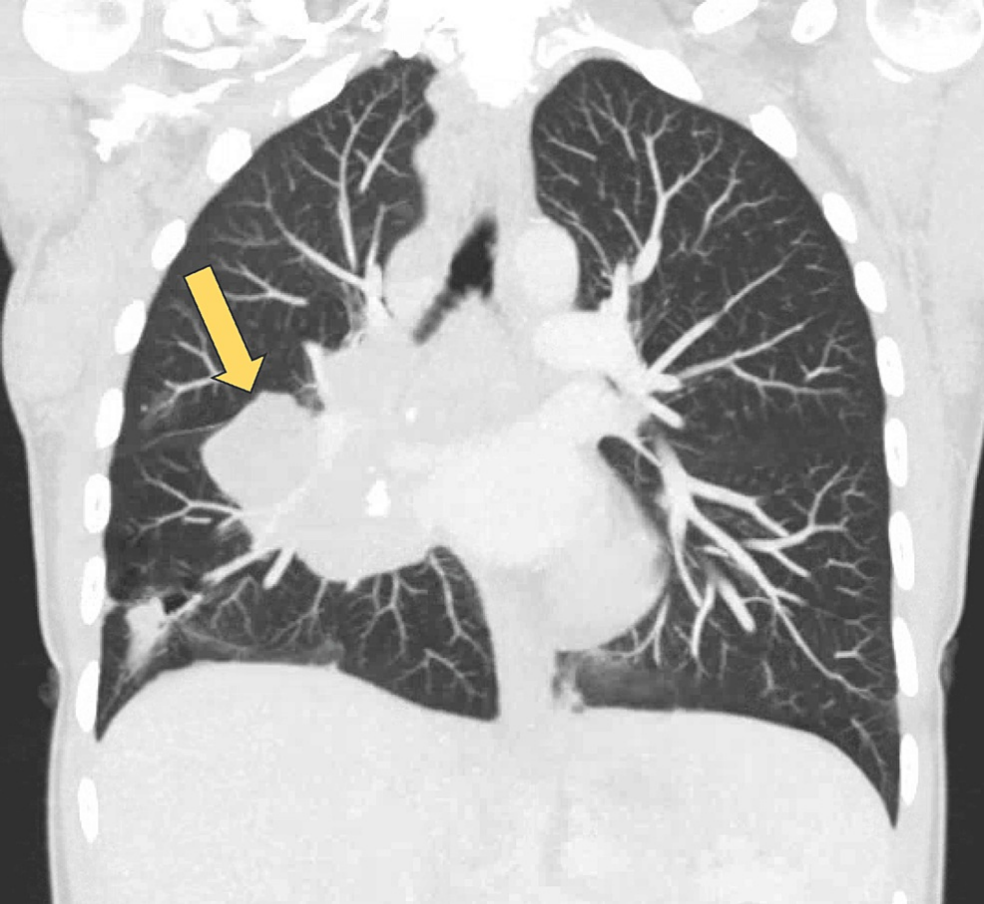
Coronal computed tomography image of the chest with contrast showing bulky right paratracheal and hilar lymphadenopathy and a right lower lobe mass.

An initial chest X-ray revealed infiltrates in the right middle and lower lobes of the lung with right-sided paratracheal lymphadenopathy. Computed tomography (CT) of the chest with contrast revealed extensive right-sided adenopathy involving the paratracheal, subcarinal, pericarinal, and hilar lymph nodes, along with hypodensities in the right lower lobe (Figure 1).

Pulmonary medicine was consulted to obtain a tissue biopsy of the lesions. She underwent endobronchial ultrasound which showed an irregular, vascular endobronchial lesion in the bronchus intermedius likely obstructing the right lower lobe. A lymph node survey showed large subcarinal and right hilar masses near the endobronchial lesion. The patient was counseled regarding concern for possible malignancy and was discharged with plans for follow-up in the oncology clinic pending biopsy results.

**Figure 2:**
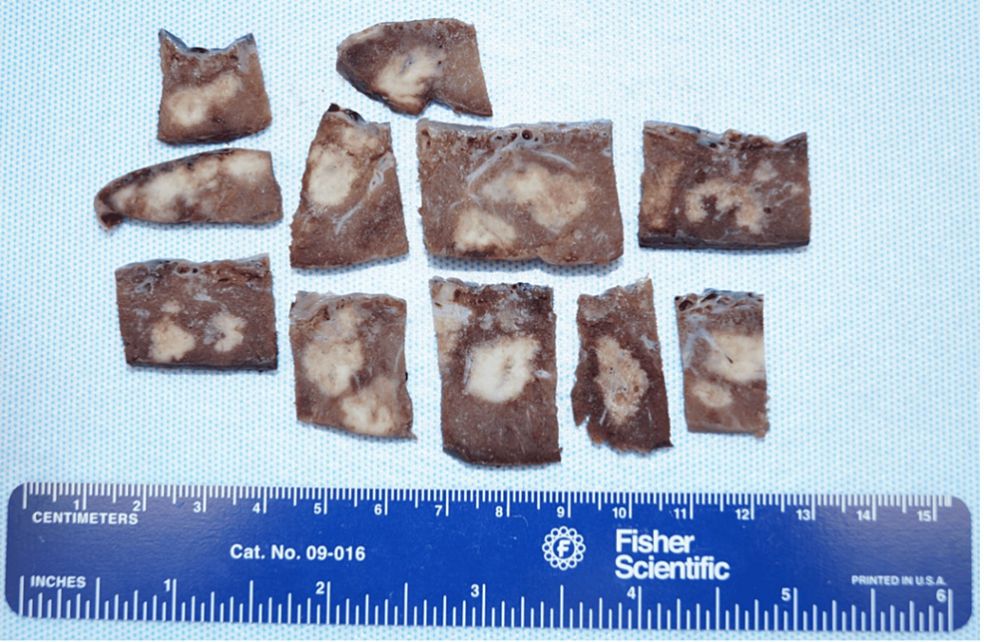
Gross pathology of the placenta demonstrating tan-colored nodular lesions.

**Figure 3:**
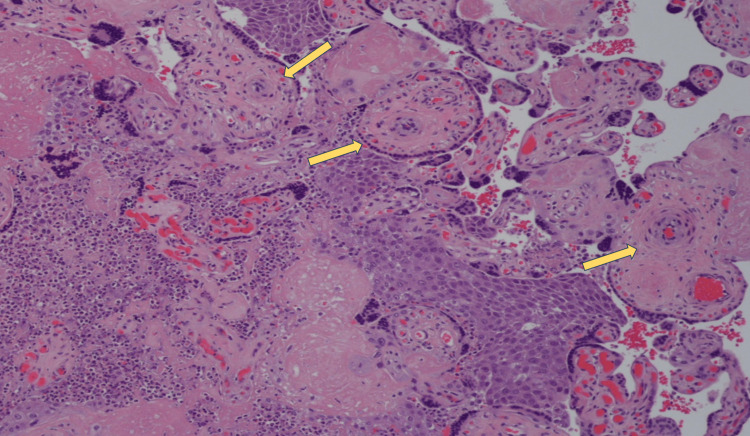
Histopathology of placental nodules showing clusters of atypical cells with squamous morphology as well as central areas of necrosis and fibrin deposition suggestive of squamous cell carcinoma.

All bronchoscopy biopsy specimens demonstrated squamous cell carcinoma. On gross examination, the patient’s placenta showed multiple tan nodules (Figure 2), and histopathology revealed multifocal regions of squamous cell carcinoma consistent with the lung biopsies (Figure 3).

The patient was diagnosed with primary squamous cell carcinoma of the lung with metastasis to the placenta. Further testing revealed that all tumor cells were 100% positive for programmed death-ligand 1 (PD-L1) expression. The patient was advised to follow up with a neonatologist to ensure the newborn was not affected by the malignancy.

**Figure 4:**
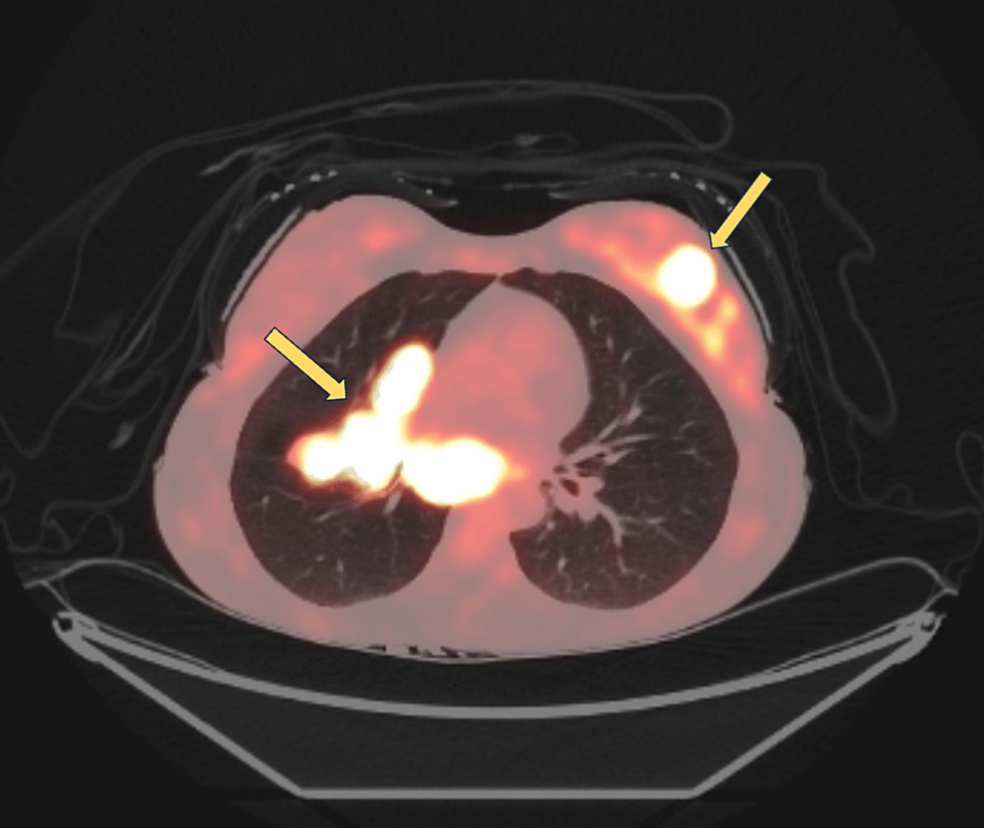
A positron emission tomography scan showing hypermetabolic regions in the right lung lobes, with avidity in the right paratracheal region, and an enhancing lesion in the left breast.

The patient’s oncologic evaluation included a staging positron emission tomography (PET) which showed hypermetabolic regions in the right middle and lower lobe of the lung, with avidity in the right paratracheal region and an enhancing lesion in the left breast (Figure 4).

The biopsy from the breast lesion was positive for squamous non-small-cell lung cancer. She was diagnosed as a Stage IVB (T1c, N3, M1c). Because of the uniform PD-L1 expression, the patient was started on immunotherapy with pembrolizumab at 200 mg intravenously every three weeks. After five cycles of pembrolizumab, a restaging PET scan was obtained. It showed a mixed response with persistent avidity in the left breast and decreased lung enhancement. Paclitaxel and carboplatin were added to gain better control over her disease. The patient missed multiple treatments and office visits due to financial concerns which delayed completion of the reconstituted immunotherapy/chemotherapy regimen. A second restaging PET scan showed an inadequate response with new lung lesions. The patient passed away before any further treatment changes could be implemented.

## Discussion

RRP is an insidious disease that progressively diminishes patient quality of life and often results in devastating long-term complications. RRP has a characteristic bimodal age distribution, presenting in children between two and four years of age and in adults between 20-40 years of age [3,9]. The incidence is 1.8 per 100,000 in adults and 4.3 per 100,000 in children [10]. JoRRP is generally a more aggressive disease than adult-onset RRP, possessing higher recurrence rates and greater risk for multifocal disease [6,9]. JoRRP additionally conveys a higher likelihood of distal respiratory tract involvement, with male patients who require frequent surgical interventions at greatest risk [8,11]. Overall, 90% of RRP cases are due to infection with HPV 6 and 11; however, serotype 11 is more closely linked to severe disease and malignant transformation [3,6,8,9]. Our patient tested negative for serotypes 1, 2, 6, 11, and 19. Unfortunately, she might have had an infection with another serotype which was not tested for. The definitive diagnosis of respiratory papillomatosis is obtained by biopsy with subsequent histopathological visualization. There is no definitive treatment for RRP and management is centered around maintaining phonation and airway patency. The symptoms of RRP are directly related to the size and number of lesions, thus surgical debulking is the mainstay of treatment with patients requiring frequent invasive procedures [9,12]. Our patient had frequent recurrences and underwent multiple papilloma excisions and biopsies. Adjuvant treatment includes intralesional therapy with cidofovir, bevacizumab, or interferon-alpha [12,13].

RRP involves the distal airway in 2-5% of patients, with disease reaching the pulmonary parenchyma in only 1% of cases [5,6]. The median duration between diagnosis of RRP and pulmonary spread is eight years [3]. Currently, the pulmonary spread is believed to be due to a combination of iatrogenic factors from repeated procedures (laryngoscopies, bronchoscopies, tracheostomies, and surgical debulking), contiguous papilloma extension, and viral dissemination [5,6]. This patient had multiple procedures such as laryngoscopies, bronchoscopies, and excision of lesions which were unavoidable but may be a potential cause of pulmonary involvement in this case. Smoking is an important factor to consider in patients with a long-standing history of chronic tobacco use [7,8] for pulmonary involvement and malignant transformation. Regular CT imaging plays an important role in the surveillance of patients for the transformation of RRP. We were unsure if our patient had previous laryngeal papilloma extension into the lungs because we lacked CT imaging of the chest before her cancer diagnosis. However, her laryngoscopic exam two months before her cancer diagnosis was negative for primary bronchial or carinal lesions.

Pregnancies complicated by malignancy occur in 1 per 1,000-1,500 gestations [14]. Fewer than 80 cases of metastasis to the products of conception (POC), i.e., fetus or placenta, have ever been reported [15]. Approximately 20% of placental metastasis is due to lung cancer, suggesting that lung cancers have a relatively high predilection for the POC [15-17]. Indeed, placental growth factors and matrix metalloproteinases have been implicated in the invasion of non-small-cell lung cancer into the placental tissues, facilitating sequestration in the intervillous space [18]. Thus, the POC should be histologically examined for metastases in all pregnancies complicated by lung cancer. Delivery should not be altered in mothers with lung cancer as most will give birth to healthy babies [16]. However, careful surveillance is crucial to intra and postpartum care of both mother and baby.

Metastasis to the breast is extremely rare [19]. Malignant melanoma is the most frequent tumor to metastasize to the breast, followed by rhabdomyosarcoma and lung cancer [20]. To our knowledge, this is the first report of squamous cell lung cancer with metastasis to the breast in a patient with RRP. Although primary breast tumors are more common, the possibility of metastatic breast lesions should not be overlooked. It is extremely important to pursue histopathological diagnosis as in the case of our patient to determine the tumor origin and formulate an appropriate treatment plan.

## Conclusions

RRP is a recurrent disease that is usually characterized by benign papillomatous lesions that can develop anywhere along the aero-digestive tract leading to life-threatening complications. Occasionally, the lung parenchyma may also be involved. RRP has malignant potential to squamous non-small-cell lung carcinoma that is increased by smoking, alcohol use, immunosuppressive drugs, frequent RRP recurrence, recurrent procedures, radiation exposure, and infection with HPV serotype 11. Frequent surveillance with CT of the chest should be considered in patients with RRP, at least in those with frequent recurrences. Although extremely rare, squamous non-small-cell lung cancer can metastasize to the placenta and even the breast. In the case of placental metastasis, careful surveillance of both mother and baby is highly recommended. There should be a high index of suspicion for a metastatic breast lesion in patients with a concurrent non-mammary primary tumor. Our case highlights the need for future research to identify the risk factors for the malignant potential of RRP to squamous cell carcinoma. This case also highlights the need to explore options for cancer surveillance and treatment for patients with RRP and high-risk features of malignant transformation.
